# Ketogenic diet sensitizes glucose control of hippocampal excitability^1^

**DOI:** 10.1194/jlr.M046755

**Published:** 2014-11

**Authors:** Masahito Kawamura, David N. Ruskin, Jonathan D. Geiger, Detlev Boison, Susan A. Masino

**Affiliations:** * Department of Pharmacology, Jikei University School of Medicine, Minato-ku, Tokyo 105-8461, Japan; † Psychology Department and Neuroscience Program, Trinity College, Hartford, CT 06106; § Department of Basic Biomedical Sciences, University of North Dakota School of Medicine and Health Sciences, Grand Forks, ND 58203; ** Robert Stone Dow Neurobiology Laboratories, Legacy Research Institute, Portland, OR 97232

**Keywords:** adenosine A_1_ receptors, bicuculline, 8-cyclopentyl-1,3-dipropylxanthine, epilepsy, K_ATP_ channel, ketones, metabolism, pannexin, purine, seizure

## Abstract

A high-fat low-carbohydrate ketogenic diet (KD) is an effective treatment for refractory epilepsy, yet myriad metabolic effects in vivo have not been reconciled clearly with neuronal effects. A KD limits blood glucose and produces ketone bodies from β-oxidation of lipids. Studies have explored changes in ketone bodies and/or glucose in the effects of the KD, and glucose is increasingly implicated in neurological conditions. To examine the interaction between altered glucose and the neural effects of a KD, we fed rats and mice a KD and restricted glucose in vitro while examining the seizure-prone CA3 region of acute hippocampal slices. Slices from KD-fed animals were sensitive to small physiological changes in glucose, and showed reduced excitability and seizure propensity. Similar to clinical observations, reduced excitability depended on maintaining reduced glucose. Enhanced glucose sensitivity and reduced excitability were absent in slices obtained from KD-fed mice lacking adenosine A_1_ receptors (A_1_Rs); in slices from normal animals effects of the KD could be reversed with blockers of pannexin-1 channels, A_1_Rs, or K_ATP_ channels. Overall, these studies reveal that a KD sensitizes glucose-based regulation of excitability via purinergic mechanisms in the hippocampus and thus link key metabolic and direct neural effects of the KD.

Glucose availability influences central nervous system physiology and pathology, and intricate crosstalk between glucose homeostasis in the brain and periphery suggests mechanistic links between brain pathologies and the increased prevalence of obesity and diabetes (1). A high fasting glucose, in the absence of any diagnosis, correlates with atrophy of the hippocampus and amygdala (2), and emerging evidence targets insulin resistance and hyperglycemia as precipitating factors (and novel therapeutic targets) for neurodegenerative disorders such as Parkinson’s (3) and Alzheimer’s disease (4). Prediabetes, even in adolescents, has recently been associated with reduced cognitive function (5), suggesting that negative effects of increased glucose do not take decades to develop.

Equally compelling evidence indicates the inverse, i.e., reduced glucose offers diverse positive neurological effects. For example, the very low-carbohydrate ketogenic diet (KD) limits available glucose (replacing lost calories with high dietary fat) and is a retrospectively and prospectively confirmed effective treatment for epilepsy (6–11). Recent studies have suggested multiple neurological benefits of the KD including multiple sclerosis, Alzheimer’s disease, and brain cancer (12, 13).

Because the KD is therapeutically beneficial, even with refractory seizures, there is intense interest in its anticonvulsant mechanisms and their relationship to its metabolic effects. It has been proposed that elevated polyunsaturated fatty acids mediate these effects, although changes in tissue fatty acid profiles and anticonvulsant activity do not correlate in many studies (14, 15). It has also been proposed that anticonvulsant effects are mediated directly by increased levels of ketone bodies (acetone, acetoacetate, β-hydroxybutyrate) produced through β-oxidation of lipids in liver mitochondria; however, blood ketones correlate poorly with seizure control in most animal and clinical studies (16–18) and do not translate clearly into changes in neuronal activity. Nevertheless, ketone esters are being developed as a means to elevate ketone levels without a drastic change in diet (19, 20). Other established metabolic effects are increased brain ATP and, as noted above, a decreased and stable glucose level (21–23). Improved seizure control has also been observed with a low glycemic index diet (24) and a modified Atkins diet (25), further suggesting the importance of reduced glucose. We proposed a glucose-related mechanism such that KDs increase adenosine, a purine nucleoside with antiseizure effects at the inhibitory adenosine A_1_ receptor (A_1_R) (12, 26, 27). Extracellular ATP is dephosphorylated rapidly into adenosine (28), thus placing adenosine squarely between KD-induced changes in metabolism and neuronal activity. Consistent with this, we found that a KD decreases spontaneous seizures due to adenosine deficiency in mice with A_1_Rs, but is ineffective in mice lacking A_1_Rs (29).

Here, we demonstrate that KD feeding decreases in vitro seizure susceptibility and sensitizes glucose-based control of excitability in the CA3 region of the hippocampus. KD feeding neither reduced excitability nor induced glucose sensitivity in A_1_R knockout mouse slices, and blocking pannexin-1 channels, A_1_Rs or K_ATP_ channels abolished these effects in slices from normal animals. The present methods may represent a useful tool for the in vitro study of KDs. Taken together, the present experiments, initiated in vivo and evaluated in vitro, link key metabolic and direct neural effects of the KD.

## MATERIALS AND METHODS

All experiments were performed in conformity with Public Health Service Policy as defined in the Institute for Laboratory Animal Research Guide for the Care and Use of Laboratory Animals, and were approved by the Trinity College Animal Care and Use Committee. All measures were taken to minimize animal discomfort. Sprague-Dawley rats and C57Bl/6 mice [wild-type or lacking A_1_R (30)] of either sex were fed standard rodent chow [control diet (CD); LabDiet 5001] or a KD (BioServ F3666) ad libitum for 13–18 days before slice preparation at age 5–8 weeks. F3666 has a fat:(protein+carbohydrate) ratio of 6.6:1 and a protein:carbohydrate ratio of 2.6:1 (31).

Standard slice preparation and recording conditions were used, similar to our previous publications (32, 33). Briefly, rats were anesthetized with isoflurane and decapitated; trunk blood was collected and centrifuged to isolate plasma; plasma was later tested for β-hydroxybutyrate (StanBio, Boerne, TX). Four to five coronal hippocampal slices of 400 μm thickness were made in ice-cold artificial cerebrospinal fluid (aCSF) with low (3 mM) or high (11 mM) glucose concentrations containing the following: 126 mM NaCl, 3 mM KCl, 1.5 mM MgCl_2_, 2.4 mM CaCl_2_, 1.2 mM NaH_2_PO_4_, 3 or 11 mM glucose, 8 or 0 mM sucrose (to balance osmolarity with the two concentrations of glucose), and 26 mM NaHCO_3_ (osmolarity 320 mOsm, pH 7.4 when saturated with 95% O_2_ plus 5% CO_2_) with a vibrating slice cutter (Series 1000, Vibratome). Slices were incubated in aCSF saturated with 95% O_2_ plus 5% CO_2_ for 30–40 min at 37°C, then kept at room temperature for 1–5 h until recording. A slice was placed on a nylon net in the recording chamber under nylon mesh attached to a U-shaped platinum frame and submerged in and continuously perfused with aCSF at a flow rate of 2 ml/min at 32–34°C. Only one manipulation was tested in each slice. Slices in all treatment conditions (CD versus KD feeding, 3 versus 11 mM glucose incubation) remained recordable out to the longest postslicing recovery incubation tested (5 h).

For extracellular recordings, medium wall (1.5 mm) capillary filament glass was pulled on a Sutter P-97 micropipette puller (Novato, CA) using a 4-cycle program, giving electrode resistances of 8–12 MΩ. The recording electrode filled with 3 M NaCl was placed in the stratum pyramidale of the CA3 region for recording population spikes (PSs) or, in some recordings, in the stratum lucidum of the CA3 region for extracellular field excitatory postsynaptic potentials (fEPSPs). A twisted bipolar insulated tungsten electrode was placed as stimulation electrode in the hilus of the dentate gyrus; stimuli were delivered at 30 s intervals. Pulse duration was 100 μs and the intensity was adjusted such that the amplitude of evoked PS responses was between 0.6 and 1.4 mV. All electrophysiological responses were recorded via an AC amplifier (World Precision Instruments) and filtered at 1 kHz. Data were digitized (16-channel A/D board, National Instruments) at a rate of 4 kHz and analyzed on-line using custom NeuroAcquisition software (Galtware, Denver, CO). All time courses of PS are moving averages of five data points (graphs in the figures show sparse markers every 3 points).

The pannexin-1 mimetic blocking peptide ^10^panx (WRQAAFVDSY, with C-terminal amidation) and its scrambled counterpart were synthesized by Biomatik. Other drugs and chemicals were obtained from Sigma. All drugs were dissolved in aCSF at 100 times the desired final concentration and applied via syringe pump upstream in the perfusion line to reach final concentration before reaching the slice chamber (28, 32). In all figures, the point indicated as the onset of drug or altered glucose application is the calculated time when the solution first begins to mix into the volume of the slice chamber. Bicuculline was applied for 20 min before subsequent treatments. Other pharmacological agents were applied for at least 15 min before subsequent treatments.

**Fig. 1.**
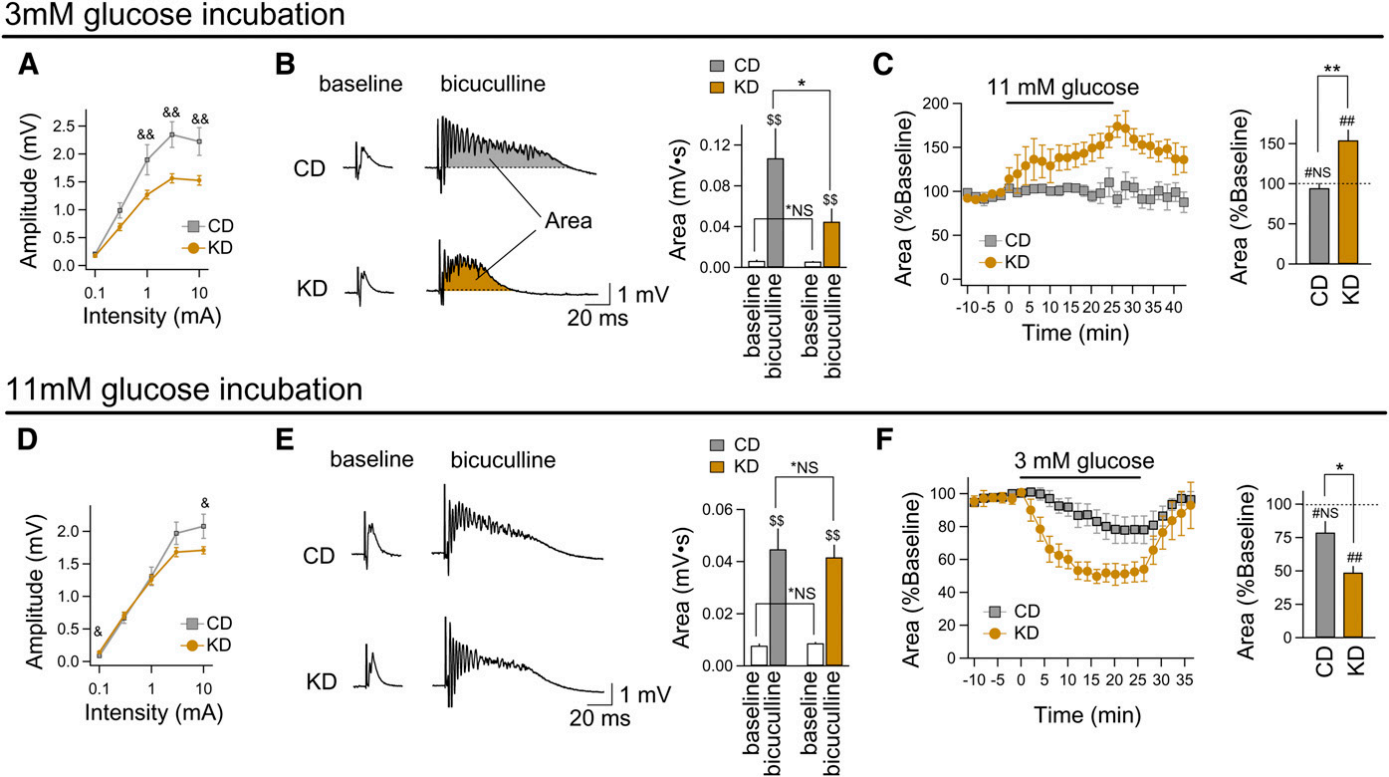
KD feeding in vivo and reduced glucose in vitro limit excitability and control seizure-like activity in rat hippocampus. A–C: Data from hippocampal slices incubated in reduced (3 mM) glucose. D–F: Data from hippocampal slices incubated in standard (11 mM) glucose. A: PS input-output curves demonstrate that hippocampal CA3 in KD-fed rats is less excitable across a range of stimulation intensities, and the maximum response amplitude was significantly lower. CD (n = 5), KD (n = 20); &&*P* < 0.01 compared between CD and KD. B: After matching for initial response amplitude, block of GABAergic inhibition (bicuculline, 10 μM) induced seizure-like activity in all slices (quantified as area under evoked response). The response area was reduced significantly in slices from KD-fed rats. CD (n = 5), KD (n = 20); *NS, not significantly different; *P < 0.05 between CD and KD; $$*P* < 0.01 between baseline and bicuculline. C: Acutely increasing glucose (from 3 mM to 11 mM) augments bicuculline-induced seizure-like activity significantly in the CA3 region of slices from KD-fed rats, but has no effect in slices from CD-fed rats. For comparability, seizure-like activity prior to acute glucose [which differed between CD and KD treatment; see (B)] is set to 100% to form new baselines for better comparison of acute glucose effects. n = 4–5; #NS, not significantly different; ##*P* < 0.01 compared with 100% (Mann-Whitney *U* test); **P < 0.01 between CD and KD. D, E: Slices from KD-fed rats incubated and recorded in 11 mM glucose showed minor electrophysiological changes in hippocampal pyramidal neurons, even during block of GABAergic inhibition. CD (n = 14), KD (n = 27); &*P* < 0.05 between CD and KD; *NS, not significantly different between CD and KD; $$*P* < 0.01 between baseline and bicuculline. F: When glucose was reduced acutely (from 11 mM to 3 mM), there was a reduction in bicuculline-induced excitability only in slices from KD-fed rats. CD (n = 13), KD (n = 7); #NS, not significantly different; ##*P* < 0.01 compared with 100% (Mann-Whitney *U* test); *P < 0.05 between CD and KD.

Recorded extracellular field potentials were analyzed off-line with NeuroAnalysis software (Galtware) and Igor Pro 5 (WaveMetrics, Lake Oswego, OR). All data are expressed as mean ± standard error. The area of the PS was measured at 20 min after bicuculline application (Fig. 1B), 15 min after other drug applications [8-cyclopentyl-1,3-dipropylxanthine (DPCPX), ^10^panx, and tolbutamide; Fig. 3], or 20 min after increased extracellular glucose concentration (Fig. 1C). The amplitude of the PS was also measured and all results of the amplitude data were the same as the results of the area (data not shown). Differences of evoked potentials with 11 mM glucose were compared with the nonparametric Mann-Whitney *U* test for normalized values. Evoked potential areas between CD and KD or between before and after drug treatment were compared with one-way ANOVA. *P* < 0.05 was considered significant.

## RESULTS

We fed a CD or KD to rats or mice for 13–18 days and prepared acute hippocampal slices for extracellular field potential recordings in CA3. Analysis of rat blood plasma indicated significant elevation of the ketone body β-hydroxybutyrate at time of euthanization (0.97 ± 0.14 mM KD vs. 0.05 ± 0.02 mM CD, *P* < 0.001). Similar and consistent changes in blood chemistry were found in mice (data not shown). Stimulation intensity was not significantly different in slices from KD-fed and CD-fed rats (0.72 ± 0.09 mA KD vs. 0.51 ± 0.13 mA CD; *P* > 0.05); also, the average adjusted PS amplitude before the application of bicuculline was not significantly different between CD and KD groups (1.00 ± 0.05 mV KD vs. 1.18 ± 0.12 mV CD; *P* > 0.05).

To maintain in vitro conditions like those in vivo during KD feeding (stable, low blood glucose), some hippocampal slices were incubated and recorded in aCSF with glucose at a low concentration (3 mM) (34, 35); other slices were incubated in high-glucose aCSF (11 mM; typical for acute slices). KD feeding reduced excitability as quantified by PS current/voltage input/output curves, particularly at higher stimulation intensities in 3 mM glucose-incubated slices (**Fig. 1A**). Furthermore, after incubation in 3 mM glucose, seizure-like activity induced by blocking γ-aminobutyric acid-mediated (GABAergic) inhibition (bicuculline, 10 μM) was diminished in slices obtained from KD-fed rats compared with those from CD-fed rats (Fig. 1B). Reduced excitability promoted by the KD was masked by 11 mM glucose incubation: compared with slices from CD-fed rats, prior KD feeding had minimal effects on the input/output relationship (Fig. 1D) and no significant effects on the area of the epileptiform discharge evoked by bicuculline (Fig. 1E) in 11 mM glucose. These results argue strongly that the effect of the KD depends on maintaining reduced glucose (3 mM). A comparison of the input/output curves of slices from CD-fed rats incubated in 3 and 11 mM glucose showed minor differences that were only significant at the lowest intensity (analysis not shown), generally consistent with the reported hippocampal PS stability during extended perfusion with 4 or 10 mM glucose (36). The dynamic influence of glucose on hippocampal excitability was selective to slices obtained from KD-fed rats. Increasing glucose to 11 mM after 3 mM glucose slice incubation increased excitability in slices from KD-fed but not CD-fed rats (Fig. 1C), similar to breakthrough seizures in KD-fed epileptic patients after carbohydrate ingestion (21). Note that in Fig. 1C, baseline levels are set to 100%, but areas were higher in slices from CD-fed rats. Conversely, reducing glucose to 3 mM after 11 mM glucose incubation decreased the response area significantly solely in slices obtained from KD-fed rats (Fig. 1F). Thus, glucose dynamically controls CA3 excitability after in vivo KD feeding.

**Fig. 2.**
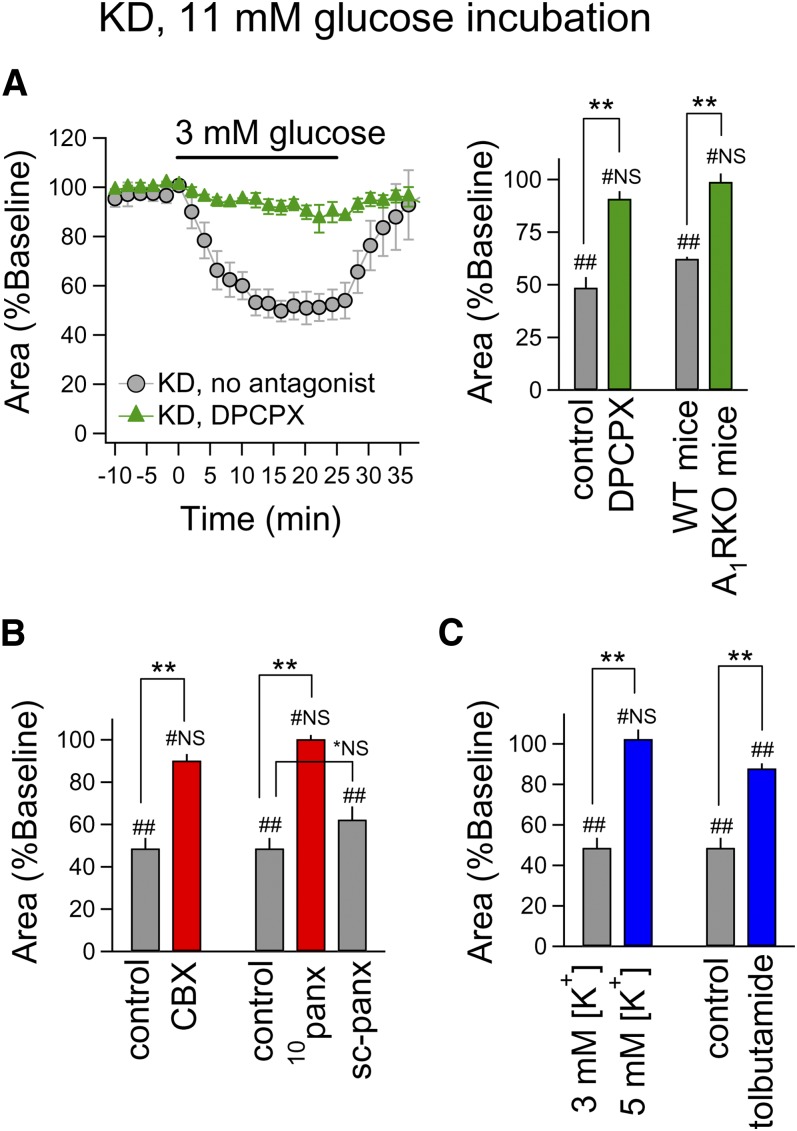
Acute glucose reduction controls the KD’s effect on hippocampal excitability via an A_1_R-pannexin-K^+^ channel pathway. All slices were incubated in 11 mM glucose aCSF and acutely switched to 3 mM glucose for 25 min as shown. Bicuculline was applied 20 min prior to other drugs or to high K^+^. A (left): Pretreatment with a selective A_1_R antagonist (DPCPX, 1 μM) blocked the inhibition of epileptiform activity due to reduced glucose in slices from KD-fed rats (n = 5–7). DPCPX itself had no significant effects on the area of seizure-like activity in 11 mM glucose-incubated slices (data not shown). A (right): After KD feeding, the inhibitory effect of reduced glucose was completely inhibited during pharmacological (rat; DPCPX) or genetic (mouse) inactivation of A_1_R. Control (n = 7), DPCPX (n = 5), WT mice (n = 5), A_1_RKO mice (n = 5); #NS, not significantly different; ##*P* < 0.01 compared with 100% (Mann-Whitney U test); **P < 0.01 between control and DPCPX or between WT and A_1_RKO mice. B: After KD feeding, the inhibitory effect of reduced glucose was blocked with antagonism of pannexin channels (CBX, 10 μM; ^10^panx, 100 μM) but not with a scrambled peptide sequence (sc-panx). Control (n = 7), CBX (n = 5), ^10^panx (n = 5), sc-panx (n = 4); #NS, not significantly different; ##*P* < 0.01 compared with 100% (Mann-Whitney *U* test); *NS, not significantly different; **P < 0.01 between control and drug. C: After KD feeding, the inhibitory effect of reduced glucose was blocked by raising extracellular K^+^ or by antagonizing K_ATP_ channels (tolbutamide, 500 μM). n = 5–7; #NS, not significantly different; ##*P* < 0.01 compared with 100% (Mann-Whitney *U* test); **P < 0.01 between 3 mM and 5 mM [K^+^] or between control and tolbutamide.

Regarding underlying mechanisms, we revealed an essential role for adenosine, an endogenous neuromodulator that links metabolism to decreased neuronal activity via A_1_Rs (37). In 11 mM glucose-incubated slices from KD-fed rats, reduced excitability upon exposure to 3 mM glucose was blocked completely by the A_1_R antagonist DPCPX (**Fig. 2A**). Additionally, KD-related reduced excitability was evident in slices obtained from wild-type mice but absent in those from A_1_R knockout mice (Fig. 2A). These data suggest that KD feeding activates A_1_R in the hippocampus. Activation of A_1_R is known to cause presynaptic reduction of glutamate input and postsynaptic increase of K^+^ conductance in CA3 pyramidal neurons (38). Interestingly, in recordings in the stratum lucidum, the amplitude of fEPSPs and paired-pulse ratios did not change with reduced glucose in 11 mM glucose-incubated slices from KD-fed rats, suggesting that these A_1_R effects are mainly postsynaptic (data not shown). Previous in vitro work has shown that, during reduced glucose, adenosine can be produced from ATP released via pannexin-1 channels and consequently reduce excitability via A_1_Rs linked to postsynaptic K_ATP_ channels (39); other studies have also implicated K_ATP_ channels in the effects of a KD (40, 41). Here, blockade of pannexin channels with a pannexin-selective dose of carbenoxolone (42) or a specific peptide antagonist, ^10^panx, eliminated effects of reduced glucose, similar to the A_1_R antagonist (Fig. 2B). Reduced excitability also depended on K_ATP_ channels: all effects were blocked by increasing extracellular K^+^ (from 3 mM to 5 mM) or antagonizing K_ATP_ channels selectively with tolbutamide (Fig. 2C).

**Fig. 3.**
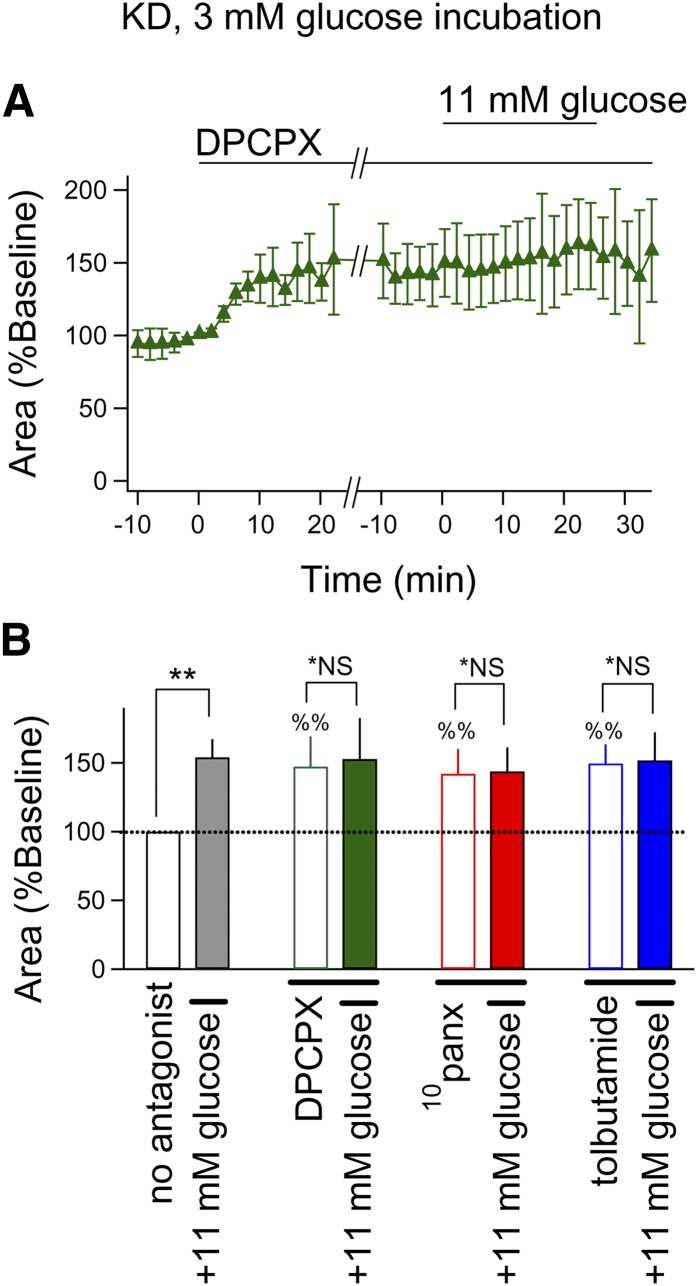
Acute elevation in glucose blocks the KD’s effect on hippocampal excitability via an A_1_R-pannexin-K^+^ channel pathway. All slices were incubated in 3 mM glucose aCSF and extracellular glucose concentration was acutely increased to 11 mM glucose for 25 min. Bicuculline was applied for 20 min before other drugs. A: DPCPX application (1 μM) augmented bicuculline-induced seizure-like activity in slices from KD-fed rats and blocked 11 mM glucose-induced increase in this activity (n = 4). B: Blocking A_1_Rs, pannexin-1 channels, or K_ATP_ channels (DPCPX, 1 μM; ^10^panx, 100 μM; tolbutamide, 500 μM, respectively) increased epileptiform activity similarly in slices from KD-fed rats. The excitatory effect of acutely increased glucose was prevented by all three antagonists. n = 4–5; %%*P* < 0.01 compared pre- and postdrug application (Mann-Whitney *U* test); *NS, not significantly different between baseline and 11 mM glucose; **P < 0.01 between baseline and 11 mM glucose.

To further explore this phenomenon, we determined the involvement of these targets on the increased excitability in CA3 produced by switching slices from KD-fed rats from 3 mM to 11 mM aCSF during recording. We found that blocking A_1_Rs, pannexin-1 channels, or K_ATP_ channels all enhanced seizure-like activity, and this enhanced activity occluded the excitatory effects of 11 mM glucose (**Fig. 3**).

## DISCUSSION

Here, we found that 2–3 weeks of KD feeding in rats and mice induced glucose sensitivity and reduced excitability in the CA3 region of acute hippocampal slices. Reduced excitability depended on maintaining reduced glucose in vitro; effects of the KD were reversed or masked by 11 mM glucose (a standard for most brain slice physiology). Reduced excitability and heightened glucose sensitivity were absent in slices obtained from mice with a genetic deletion of A_1_Rs and abolished in slices during a pharmacological blockade of A_1_Rs, pannexin-1 channels, or K_ATP_ channels. Because A_1_Rs couple to K_ATP_ channels to reduce postsynaptic excitability, these experiments identify lowered glucose and elevated A_1_R activity as key links to specific neuronal mechanisms of the KD, and suggest a new experimental preparation, in vivo KD feeding followed by reduced glucose in vitro, for further study of the KD.

Even though we lowered extracellular glucose, we observed no signs that slices incubated in 3 mM glucose were significantly hypoglycemic. Hypoglycemia is well-known to release adenosine (43), which would have driven the input/output curve downward compared with slices incubated in 11 mM glucose; such a change in the curve did not occur. Slices incubated in 3 mM glucose (and 11 mM) remained similarly recordable and thus apparently healthy out to our maximum slice recovery time of 5 h. In a previous study using identical slicing and recording conditions in the identical hippocampal substructure (in tissue from CD-fed animals), we presented data inferring that adenosine tone at A_1_Rs was similar in 3 mM and 11 mM glucose. The A_1_R antagonist DPCPX alone reduced tonic outward (K^+^) current mildly in 11 mM glucose; when applied after ∼20 min of 3 mM glucose, DPCPX reduced outward current to a virtually identical extent [compare Fig. 3A “pretreatment” DPCPX vs. “reversal” DPCPX in (39)]. Adenosine tone is thus similar in both conditions; therefore, significant hypoglycemia is unlikely in our particular experimental parameters.

It is well-established that the brain regulates glucose metabolism (1) and that glucose can influence seizures (21). Our findings are consistent with research observations that blood glucose level can correlate directly with seizure frequency (44, 45), and observations that anticonvulsant effects of the KD in vivo reverse quickly upon glucose injection or by ingesting carbohydrate-rich food in both animal models of epilepsy (29, 46, 47) and epileptic patients (21). Thus, KD feeding sensitizes hippocampal circuitry to changes in glucose whereby: *1*) maintaining reduced glucose (3 mM) in acute hippocampal slices in vitro sustains the reduced excitability promoted by the KD in vivo; and *2*) elevating glucose (11 mM) models the breakthrough seizures in patients on a KD who ingest carbohydrates. Based on these findings, limited consequences of KD feeding in prior experiments with acute in vitro slices might be due to incubation and superfusion with 11 mM aCSF glucose: we found minimal changes in the input/output relationship when slices from KD-fed animals were recorded in standard aCSF.

In a prior study, we modeled a KD in vitro by acutely lowering extracellular glucose and maintaining or elevating intracellular ATP in CA3. Under these metabolic conditions, designed to mimic a KD, we also demonstrated inhibitory effects in pyramidal neurons mediated by A_1_Rs linked to K_ATP_ channels, an effect that was not present in astrocytes (39). However in these previous experiments we did not use a dietary treatment: their focus was on establishing metabolic endpoints of the diet. Accordingly, our approach was similar to other studies using in vitro electrophysiology to increase understanding of neural mechanisms underlying the effects of ketone-based metabolism, for example, by applying ketones in vitro (40, 48–50). Whereas in vitro manipulations can offer exact control over experimental variables and elucidate mechanisms, overall they lack a connection to the diverse metabolic changes that occur in vivo with a dietary treatment.

Here, after KD feeding for several weeks, the cohort of mechanisms described with our acute in vitro model of the KD was recapitulated. The A_1_R-based control of excitation observed here is consistent with the KD’s effects quantified in vivo in transgenic mice with electrographic seizures due to adenosine deficiency (29). Elevation of adenosine and heightened activation of A_1_Rs could explain the KD’s anticonvulsant success against a wide range of seizure disorders (26), because A_1_R activation is effective in virtually every tested animal model of seizures (51) including pharmacoresistant seizures (52). To date, an established model of the KD in vitro has never been established; a recent paper examining CSF from mice fed a KD helps address this knowledge gap (53), and we suggest that the match among the present experiments, previous in vivo experiments (29), and our metabolic mimic in vitro (39) suggest that reduced glucose and sufficient ATP are critical in mobilizing adenosine-based anticonvulsant effects.

Interest has intensified recently toward understanding key mechanisms underlying the KD’s anticonvulsant effects and, to that end, in establishing an effective protocol to assess in vitro the effects of KD feeding. This interest is due to increasingly widespread and international use of the KD for epilepsy and, in parallel, a burgeoning interest in metabolic approaches as a platform for new therapies for diverse neurological disorders. Overall, the present experiments represent the first study delineating processes mobilized in vivo by KD feeding that: *1*) link to and depend on known metabolic effects (limited glucose); *2*) identify specific anticonvulsant neuronal mechanisms, i.e., reducing excitability in a seizure-prone area via pannexin-1 channels, adenosine A_1_Rs, and ultimately K_ATP_ channels; and *3*) as observed clinically, reverse with increased glucose.
